# Life-threatening cardiac episode in a Polish patient carrying contiguous gene microdeletion of the *TBX5* and the *TBX3* genes

**DOI:** 10.1186/s40064-016-3275-1

**Published:** 2016-09-21

**Authors:** Katarzyna Iwanicka-Pronicka, Magdalena Socha, Maria Jędrzejowska, Małgorzata Krajewska-Walasek, Aleksander Jamsheer

**Affiliations:** 1 Department of Medical Genetics, Children’s Memorial Health Institute, Al. Dzieci Polskich 20, 04-930 Warsaw, Poland; 2 Department of Audiology and Phoniatrics, Children’s Memorial Health Institute, Al. Dzieci Polskich 20, 04-930 Warsaw, Poland; 3 Department of Medical Genetics, Poznan University of Medical Sciences, Fredry 10, 61-701 Poznan, Poland; 4 NZOZ Center for Medical Genetics, GENESIS ul. Grudzieniec 4, 60-601 Poznan, Poland

**Keywords:** Microdeletion, Array-CGH, Holt–Oram syndrome, Ulnar-mammary syndrome, Heart block, TBX5 gene, TBX3 gene

## Abstract

Holt–Oram syndrome (HOS) features radial ray hypoplasia, heart defect and cardiac conduction impairment. Ulnar-mammary syndrome (UMS) characterizes congenital defects of the ulnar side of the upper limbs, underdevelopment of apocrine glands including hypoplasia and the dysfunction of mammary glands, hypogonadism and obesity. Inheritance of both conditions is autosomal dominant, mutations or deletions are found in the *TBX5* and *TBX3* gene, respectively. The Polish patient presented short stature, obesity, congenital malformation of the radial and ulnar side of the upper limbs, heart block, hypogonadism and dysmorphic features. At the age of 13 years he lost consciousness developing respiratory insufficiency caused by bradycardia in the course of sudden atrioventricular third degree heart block requiring immediate implantation of pace maker-defibrillator device. Microdeletion of the 12q24.21 was identified using array CGH method. This region includes contiguous genes the *TBX5, TBX3*, and part of *RBM19.* The patient initially diagnosed as having HOS, was found to present the UMS features as well. Array CGH method should be applied in patients suspected of HOS or UMS, especially when sequencing of *TBX5* or *TBX3* genes fails to identify causative mutation.

## Background

Holt–Oram syndrome (HOS, OMIM 142900) is a genetic condition characterized by radial ray hypoplasia and congenital heart defect often associated with progressive arrhythmias and dysmorphic features (Holt and Oram 1960; Harris and Osborne 1966). The syndrome is usually caused by point mutations or intragenic deletions of the *TBX5* gene, however in rare cases it results from larger deletions encompassing the entire *TBX5* coding sequence (Basson et al. 1997; Gruenauer-Kloevekorn and Froster 2003; Newbury-Ecob et al. 1996). Ulnar-mammary syndrome (UMS, OMIM 181450) is a congenital malformation syndrome characterized by the ulnar hypoplasia of the upper limbs, usually comprising absent or hypoplastic 5th and/or 4th fingers, absent or hypoplastic ulna, underdevelopment of the apocrine glands resulting in the dysfunction of the mammary and axillary glands, hypogonadism with genital anomalies and delayed puberty in males, obesity, absence of axillary hair and tooth abnormalities (Schinzel 1987; Pallister et al. 1976). UMS also referred to as Schinzel syndrome or Pallister ulnar-mammary syndrome can result from either point mutations or deletions of the *TBX3* gene (Bamshad et al. 1997; Linden et al. 2009). Microdeletions of the 12q24.21 region comprising the two neighbouring *TBX5* and *TBX3* genes lead to the phenotype characterized by a combination of the clinical symptoms associated with both disorders (Borozdin et al. 2006; Alby et al. 2013; Bogarapu et al. 2014).

## Case description

This boy was born at term, by Caesarean section due to foetal bradycardia. His birth weight was of 2700 g, length of 51 cm, and Apgar score of 2-2-2-7 points at 1, 3, 5, 15 min, respectively. The early psychomotor and intellectual development was normal and the boy attended public school. His parents, younger sister and two maternal half-siblings are healthy.

**Fig. 1:**
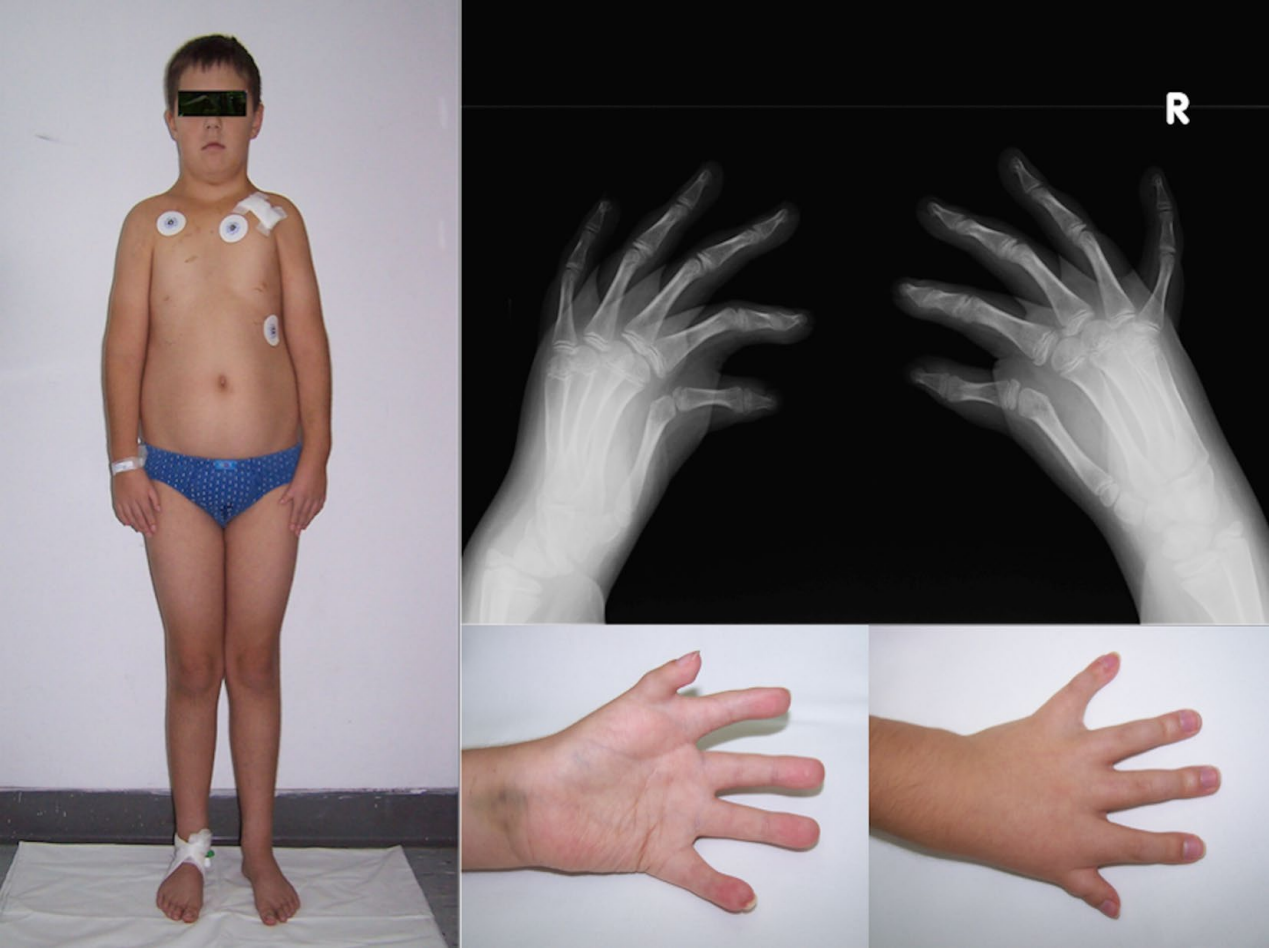
The patient’s silhouette. Rtg of the patient’s hands. Anterior and posterior palmar surface of the left hand of the patient

He was referred to our Institute at the age of 14 years due to dysmorphic features, congenital malformations of the upper limbs, heart block, and hypogonadism. The boy presented with short stature 145 cm (<3 pc), prominent truncal obesity 48 kg (25–50 pc), steatomastia, and hypoplastic, low set nipples. His neck was short with lower hairline and downsloping shoulders Fig. 1. The face was oval in shape with fat accumulation under the chin. Dysmorphic features included hypertelorism, epicanthal folds, slanting downwards palpebral fissures, long nose, long philtrum and small mouth with narrow upper lip set of the horseshoe shape. The ears were slightly protruding with thick lobes and recurvate helix. Anomalies in the oral cavity included high-arched palate and overlapping, crowded teeth. Additionally, he had small hypoplastic penis embedded in adipose tissue, right-sided cryptorchidism, underdeveloped, shawl scrotum containing a small left testicle.

Skeletal malformations comprised hypoplasia of pectoral muscles, shortening of forearms, bilateral hypoplasia of radial side of the hands (with small, hypoplastic biphalangeal yet non-opposable thumbs), hypoplasia of thenar and hypothenar eminences and brachydactyly Fig. 1. Radiologic examination revealed 3-years delay in carpal ossification Fig. 1. Echocardiography revealed mitral valve regurgitation without any structural abnormalities. At age of 13 years the boy suddenly developed cardiac arrhythmia leading to unconsciousness and rapidly progressive lung oedema. He required mechanical ventilation. This episode was followed by the development of third degree heart block resulting in severe bradycardia about 47 beat per min and prolonged Q-T interval requiring implantation of endocavitary cardioverter defibrillator device.

**Fig. 2:**
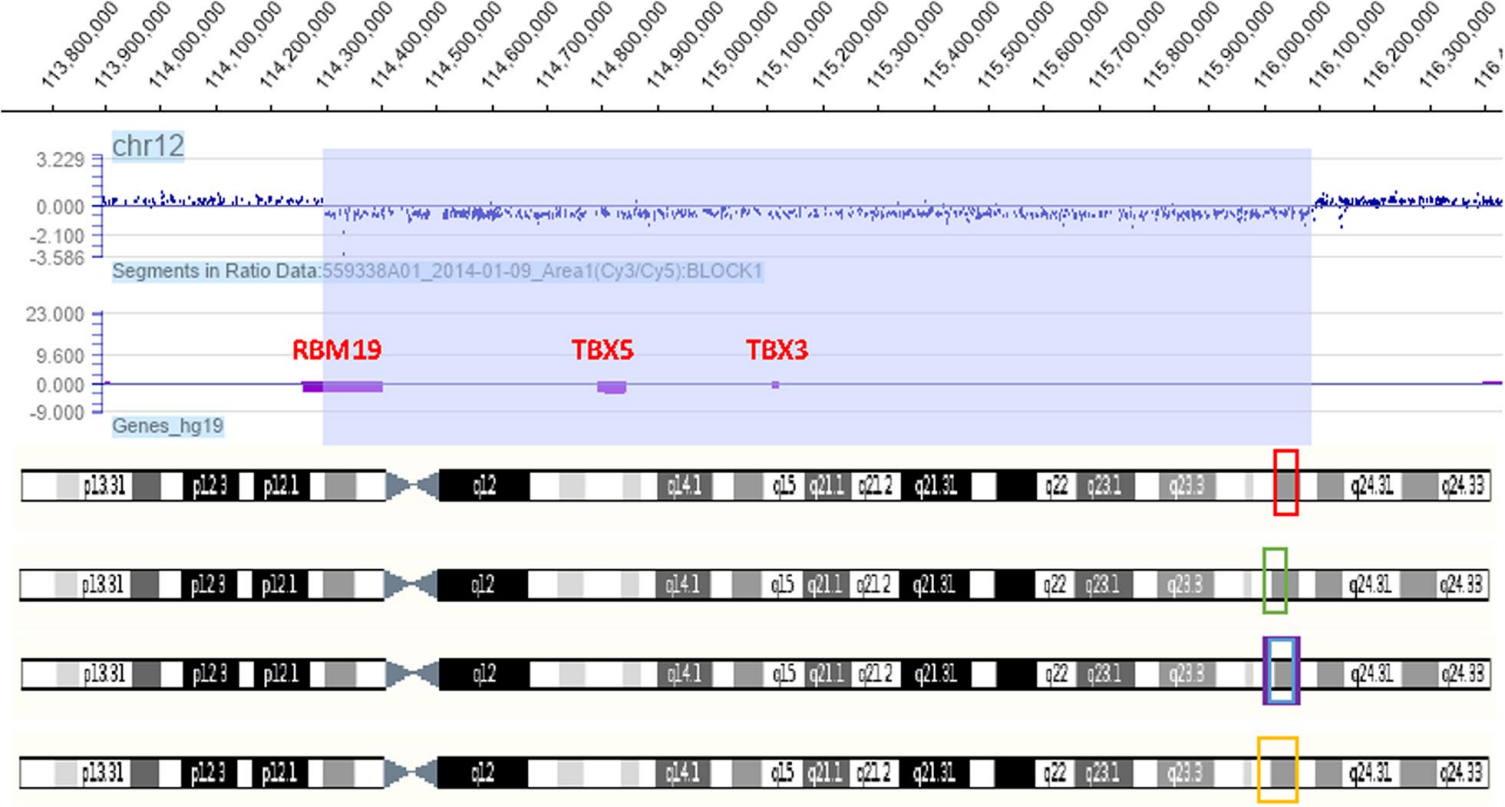
Array CGH profile of chromosome 12. *Red frame* at the chromosome 12 (at the *bottom* of the picture) localised in the region 12q24.21 indicates the microdeletion of 1.79 Mb found in the Polish proband, encompassing the contignous genes: TBX5, TBX3, and part of the RBM19; the minimal genomic coordinates 12: 114297717–116091603—(hg19). *Green frame* shows the microdeletion of 2.2–2.3 Mb found in Borozdin’s Czech family: telomere end: 114,750,000–114,800,000; centromere end: 112,560,000–112,460,000). *Blue* and *violet frames* point the microdeletions of 2.44 and 2.6 Mb found in French patients of Alby 12: 113,084,817–115,681,244—(hg19) and 12: 113,197,408–113,736,198—(hg18). *Orange frame* displays the microdeletion of 2.6 Mb found in the Spanish boy presented by Bogarapu 12: 112,963,559–116,095,198—(hg19)

Based on the phenotypic presentation and negative sequencing of the *TBX5* gene, high-resolution array CGH method was applied. Heterozygous interstitial contiguous gene deletion of the *TBX5* and *TBX3* encompassing 1.79 Mb of the chromosome 12q24.21 region was revealed and subsequently confirmed by qPCR. The minimal genomic coordinates of the detected deletion were 114297717–116091603 according to hg19 database Fig. 2. Paternal origin of the deletion was excluded with the use of qPCR, while maternal DNA was unavailable for testing.

## Discussion and evaluation

We describe a Polish proband presenting the overlapping phenotype of HOS and UMS, resulting from contiguous microdeletion comprising the *TBX5*, *TBX3* and a part of *RBM19* genes, located on chromosome 12q24.21. To date, similar molecular defects and clinical phenotypes have been described only in seven patients from four unrelated families. The size of the microdeletions varied among the patients, although our case carrying smaller than previously described changes presented the most severe, life-threatening cardiological symptoms and dysmorphia.

The first description of HOS and UMS overlap was provided by Borozdin et al. (2006), who presented a Czech family: the mother and her two daughters. A size of the microdeletion identified in those patients was delineated as 2.19 to 2.27 Mb, slightly larger than in our patient. Distinct dysmorphic features of those patients compared to our proband included hypertrichosis of the hands, back, and genital region, cleft between 4th and 5th fingers and congenital heart defect (VSD and ASD). The most serious medical problem described in one child from the Czech family involved subglottic laryngeal stenosis requiring long-term ventilation through laryngeal tube.

The next report of Alby et al. (2013) presented two unrelated French females and a foetus, all with contiguous deletions encompassing 2.6 and 2.44 Mb also involving the *TBX5* and *TBX3* genes. Both women manifested mild clinical features being a combination of both syndromes. One of them has additionally growth hormone deficiency, while her foetus presented with VSD and hypoplastic aorta.

The latest paper of Bogarapu et al. (2014) described a boy of Spanish origin, who presented with social impairment, speech and motor delay. Physically, the boy fulfilled combined diagnostic criteria of HOS and UMS. Array CGH study performed in this patient revealed the presence of two deletions: one 3.1 Mb in size encompassing *TBX5, TBX3* and 17 other genes, and the second occurring at 12p13.33 and involving four genes. Since little is known about the function of the remaining haploinsufficient genes, one can hypothesize on their possible contribution to the development of the clinical and behavioural phenotype of this patient.

The *RBM19* gene was found to be partially deleted in our proband. Literature data suggests the role of *RBM19* in the proliferation, differentiation, and development of the intestinal epithelium (Lorenzen et al. 2005). Although the impact of the partial deletion of this gene on phenotype is unknown, one can assume the substantial effect on phenotype of *RBM19* haploinsufficiency, as well as other noncoding and enhancing regions for distal genes affected by partial deletion, or microRNA fragments potentially existing in non-coding sequences of the region.

The co-occurrence of cardiac involvement is more characteristic for deletions of the *TBX5* gene that code a transcriptional activation factor promoting heart development, especially cardiac primordial structures (Bruneau et al. 2001). However, a dysfunction of heart conduction system may also result from the *TBX3* deletion (Hoogaars et al. 2007). Borozdin et al. (2006) suggested that the deletion of the *TBX3* gene may probably enhance the severity of the cardiac phenotype caused by haploinsufficiency of the *TBX5* gene. We believed that bradyarrhythmia observed in our patient is caused by *TBX5* deletion, and is not enhanced by *TBX3* rearrangement.

It was described that *TBX5* and *TBX3* evolutionary derive from the common ancestral gene, and each of them acquired a complementary role in the development of mammalian upper limb (Boehme and Shotar 1989). A diversity of symptoms encountered in patients with the deletion of the *TBX5* and *TBX3* genes reflects their versatile developmental role, involving the formation of heart, radial and ulnar side of forelimb, lungs, pharynx, thorax, body wall, and mature retina (Borozdin et al. 2006). It should be stressed that the clinical features of patients carrying the rearrangement result not only from the lack of the deleted genes, but also from other existed undetermined distal enhancer elements, or microRNAs as well.

## Conclusions

The patient carrying a contiguous microdeletion of *TBX5* and *TBX3* genes displays features of Holt–Oram Syndrome (HOS) and ulnar-mammary syndrome (UMS). The most prominent symptoms observed in our case are progressive arrhythmia leading to heart failure accompanied by heart defect, bilateral upper limb malformation of the radial and ulnar side, short stature, obesity, hypogonadism causing delayed puberty, hypoplastic apocrine glands, teeth anomalies and dysmorphia. Such molecular defect may be detected using array CGH method. The size of the microdeletion encompassing the region of 12q24.21 does not strictly influence the severity of the phenotype. Polish patient presenting with the most severe clinical manifestation in reference to the previously described patients, carries the smallest molecular change. Numerous health complications seen in patients with haploinsufficiency of the *TBX5* and *TBX3* genes entail a multidisciplinary care. Critical heart complication in our case suggests need for thorough cardiological monitoring, because of risk for sudden heart insufficiency resulting from the progressive conduction impairment. Affected newborns showing respiration problems should be suspected of having subglottic laryngeal stenosis, thus they require immediate phoniatric management.

## Abbreviations

HOS: Holt–Oram syndrome
UMS: ulnar-mammary syndrome = Schinzel syndrome or Pallister ulnar-mammary syndrome
aCGH: array comparative genomic hybridisation
